# Tutor of Resilience: A Model for Psychosocial Care Following Experiences of Adversity

**DOI:** 10.3389/fpsyt.2021.559154

**Published:** 2021-03-23

**Authors:** Francesca Giordano, Alessandra Cipolla, Michael Ungar

**Affiliations:** ^1^Department of Psychology – Resilience Research Unit, Università Cattolica del Sacro Cuore Largo Gemelli 1, Milan, Italy; ^2^Canada Research Chair in Child, Family and Community Resilience, Resilience Research Centre Dalhousie University, Halifax, NS, Canada

**Keywords:** resilience, tutor of resilience, psychosocial care, training program, adversity, service providers, children, humanitarian setting

## Abstract

This article describes a model for training service providers to provide interventions that build resilience among individuals who have experienced adversity. The Tutor of Resilience model emphasizes two distinct dimensions to training: (1) transforming service providers' perceptions of intervention beneficiaries by highlighting their strengths and capacity for healing; and (2) flexibly building contextually and culturally specific interventions through a five-phase model of program development and implementation. Tutor of Resilience has been employed successfully with child and youth populations under stress in humanitarian settings where mental health and psychosocial support professionals are required to design and deliver interventions that enhance resilience among vulnerable children.

## Introduction

Mental Health and Psychosocial Support (MHPSS) in humanitarian settings refers to “any type of local or outside support that aims to protect or promote psychosocial well-being and/or prevent or treat mental disorder” (1). This field of practice addresses the mental health issues of people in complex humanitarian emergencies by emphasizing the interaction between individual emotional wellbeing and an individual's social-ecological context (2). A large body of studies confirm that psychosocial interventions help individuals exposed to adversity to achieve positive outcomes (3–5). In particular, studies demonstrate the efficacy of adopting approaches that build resilience through psychosocial programs in humanitarian settings with population dealing with adversity associated with war (6–9) and natural disasters (10, 11).

While resilience has been traditionally thought of as a psychological trait, more recently it has been conceptualized as a dynamic developmental process that involves drawing on both internal and external resources to achieve positive outcomes despite adversity (12, 13). These processes are facilitated by protective relationships (14, 15) and supportive social and physical ecologies that make resources available and accessible in ways that individuals experience as meaningful (16). Whether formal or informal, a supportive relationship has been shown to exert a positive effect on psychological and behavioral outcomes, especially for children and youth (the focus of this paper) living in situations of atypically high risk exposure (17, 18). Indeed, even formal service providers who are trained to offer a safe, stable, and encouraging professional relationship with program beneficiaries can enhance a child's resilience over time (19, 20).

In line with this, Pillar Three, Standard 14 of the Child Protection Minimum Standards (CPMS) developed by members of the Alliance for Child Protection in Humanitarian Action (21), a coordinated response to prevent and respond to child protection challenges, highlights the importance of applying a “socio-ecological” approach that includes a focus on the needs of children, families, communities and service providers as mutually dependent parts of the child protection system. Specifically, developing and implementing child protection capacity-building initiatives to strengthen the ability of the social service workforce is essential to contextualizing and adapting evidence-based interventions in different environments.

Ensuring programming is contextually and culturally relevant, however, has been challenging for developers of interventions that build resilience (22). It this article we describe an approach to program development for mental health and psychosocial support (MHPSS) that can increase the skills of professional and non-professional helpers to act as resilience-enablers and increase beneficiaries' psychosocial well-being through well-designed interventions.

## The Tutor of Resilience Model

The Tutor of Resilience (ToR) model was developed to guide local service providers such as social workers, educators, psychologists and other helping professionals in the creation of a culturally and contextually sensitive approach to provide MHPSS in ways that both mitigate risk and enables access to resilience-promoting resources for a specific child, youth or family population experiencing one or more forms of adversity. This approach is built on the premise that resilience is a social ecological process that helps individuals navigate and negotiate for personal and collective resources through interpersonal relationships that increase access to psychosocial and physical (e.g., housing, transportation, safety, etc.) supports (16, 23).

While ToR is meant to build resilience and enhance the mental health of the focal population, conceptualized as not just the absence of mental disorder, but as a state of well-being in which every individual realizes his or her own potential, can cope with the normal stresses of life, can work productively and fruitfully, and is able to make a contribution to her or his community (24), it is an intervention for local mental health care providers themselves that familiarizes them with the principles and gives them the tools necessary to create evidence-informed resilience-enabling practices tailored to the varied contexts where they work. The model avoids one-size-fits-all solutions to programming, recognizing that populations distinguished by cultural, economic, religious and political differences require very different types of activities associated with well-being in stressed environments. Furthermore, ToR relies on the community-based psychosocial approaches in humanitarian settings that promote the involvement of local communities in all stages of MHPSS responses as they are considered the drivers for their own care and catalysts for social transformation (21). Therefore, where other resilience-promoting interventions train staff to deliver high fidelity programming slightly adapted to each context (25), ToR provides facilitators with a set of core principles that guide them in selecting, adjusting and/or tailoring the specific activities they will employ in their community-based programming to enhance engagement in resilience processes by their target beneficiaries. These principles include:

1. Widen the participants' point of view on the beneficiaries, in order not to limit it to impairments and psychological wounds but to focus attention on the beneficiaries' strengths (26–30).
2. Help beneficiaries discover their own internal resources and talents and reinforce them. In particular, the following resources have been taken into consideration and are amenable to influence through a well-designed ToR program: self-efficacy (31, 32); self-awareness (33); projecting oneself into a meaningful future (33, 34); coping abilities (35–37); and social skills (38–40).
3. Enhance beneficiaries' emotional competence (41–43) and emotional regulation (44–47) in order to mitigate negative consequences of stress (48) and decrease emotional reactivity (49) which may have an adverse effect on beneficiaries' psychosocial development (39, 50).
4. Reinforce beneficiaries' relationships with peers (51, 52), and service providers (53–58) to help develop trust in others.
5. Strengthen family systems by enhancing family cohesion (36, 59) and communication (52, 60), creating stronger family support networks (51, 61, 62) that improve caregiving (63–65).

To ensure that these core principles will be effective in real-world settings, ToR is based on findings from several large multisite action-research studies and interventions, in national and international contexts, aimed at broadening our understanding of culturally significant protective factors and processes that foster resilience. Studies have focused on populations affected by war and forced migration (66), natural disasters (48, 67), abuse and maltreatment (39, 68) and other types of adversity (69).

The ToR model proposes a twin-track approach which includes: (1) transforming the attitudes of services providers to better perceive the strengths of beneficiaries, rather than training service providers as program facilitators (70); and (2) flexibly building contextually and culturally specific interventions through a five-phase method that ensures programming will influence positively the well-being of program beneficiaries.

## Transformation, not Training

The ToR model offers service providers guidance in critical self-reflections and transformative learning, which requires creative deliberation and changes to how they conduct their practice and their understanding of resilience, shifting from a focus on individual change to a social-ecological process definition of resilience which enables better interactions between individuals and supportive environments. Among the most important and transformative aspects of training provided to ToR facilitators is encouragement to challenge deficit-based perspectives and deepen their understanding of a strength-based reframe of the behaviors of program participants (71). This immersive period of reflection encourages program leaders to take more active responsibility for self-improvement (72) by increasing their essential skills as resilience-enablers while motivating them to actively engage program participants in a similarly transformative process.

This process is carried out through the following actions:

1) A multi-day capacity building workshop that facilitates the trainees' self-confrontation and group reflection about the factors and processes that support resilience, using trainees' own experiences, thoughts, values and insights as catalysts for case studies and theory development (e.g., reflecting on the question, “What does being a Tutor of Resilience mean locally?”).
2) Following the workshop, finalization of an action plan and implementation of a resilience-enabling curriculum in the host community and a sequence of activities that are locally relevant which reflect the trainees' shift in perspective from deficit-focused thinking to resilience-enhancing processes.
3) During a follow-up workshop 3–5 months later, review the trainees' implementation of their programming, with an emphasis on drawing meaning from their experience. This is followed by an opportunity to revise and if necessary, adjust the action plan and sequence of activities to increase their impact on intervention beneficiaries.

Over the course of this training and implementation cycle, five phases of work are undertaken which reflect the principles of the ToR model.

## A Five Phase Method for Building Contextually Specific Interventions

**Figure 1:**
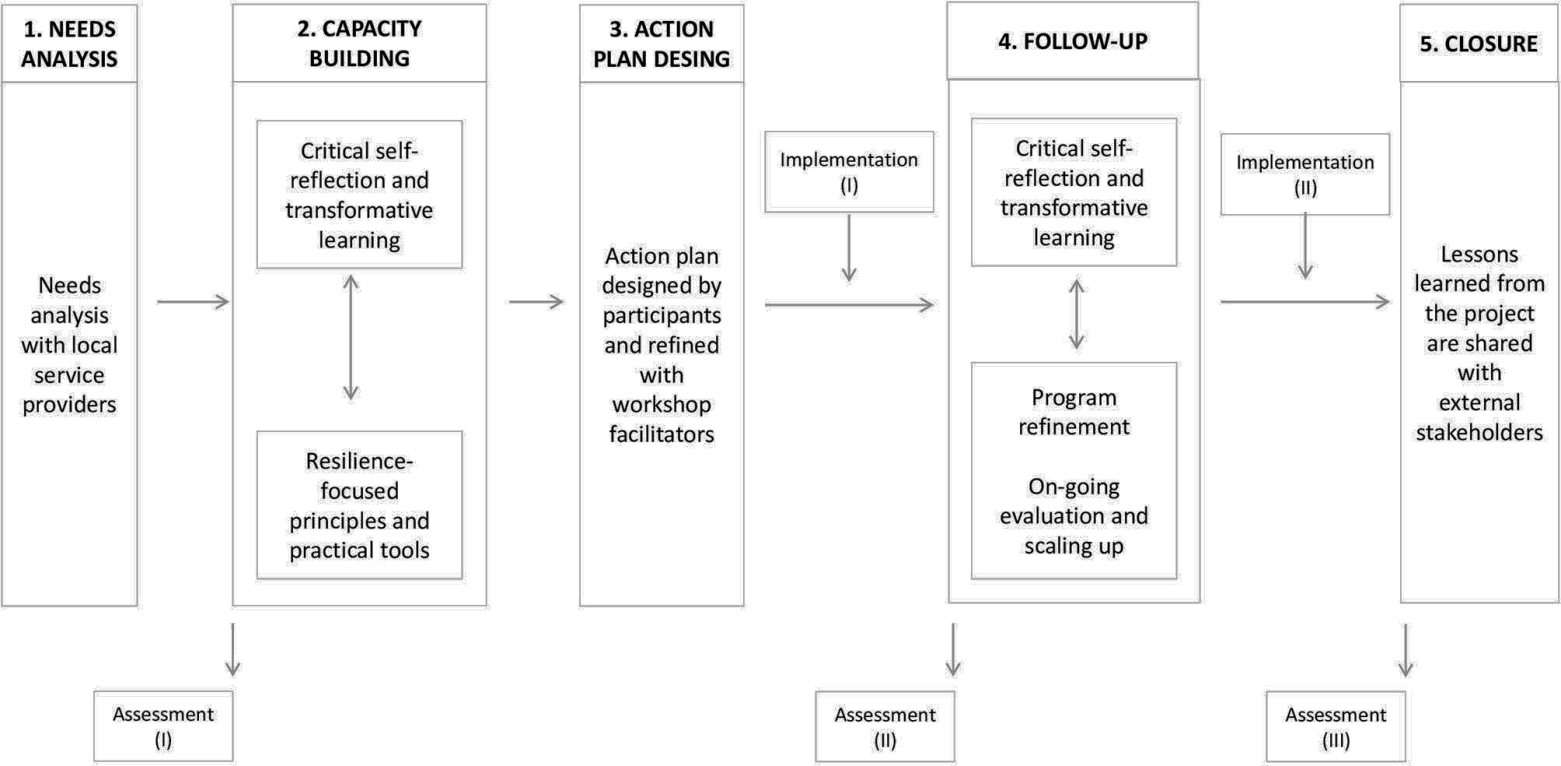
The five phases of the ToR model.

Treatments to enhance the well-being of vulnerable populations that are designed for one context and exported to another may show reduced effectiveness if they are not well-adapted to people's risk exposure or made contextually and culturally relevant (73). Explicit co-design processes— in which trainers specify major core components and an overarching structure but collaborate with participants to define more specific aspects of the intervention in real time— is proposed as a method for the development of contextually appropriate practices (74, 75). The ToR model was developed specifically to avoid these challenges which are typical of standardized programming. Its five phases (see Figure 1) ensure better integrated programming that is evidence-informed. These five phases include a needs analysis, capacity building, action plan design, follow-up and closure, with periodic assessments of outcomes to ensure intervention effectiveness.

### Phase 1: Needs Analysis With Local Service Providers

To initiate the ToR model, ToR leaders (among them the lead authors of this paper) and local co-facilitators begin work with groups of key actors drawn from local service providers. This group usually numbers between 15 and 50. These meetings have as their goal to define the psychosocial needs and barriers to service experienced by the intended beneficiaries of programming. Concurrently, trainees are polled on the most relevant protective factors and processes that could support beneficiaries in dealing with local challenges. A specific tool–Caught in a Thunderstorm (76)–is used during this stage. The risks/challenges/fears that trainees identify are written inside pictures of clouds drawn at the top of pieces of paper. For each cloud, trainees then explain in both written form and through group discussion what and/or who has helped them deal with each perceived barrier to well-being. These are drawn as umbrellas at the bottom of the page. A process of personal reflection and small group and whole group reporting ensures that consensus is reached on the most important challenges and sources of resilience, much as a Delphi process (77) encourages stakeholders to prioritize issues in a community.

### Phase 2: Capacity Building

ToR capacity building is delivered in small groups of up to 25 trainees through an initial four-day workshop where the following topics are discussed:^1^

- *Module 1: Psychosocial approaches to promote and maintain resilience*. Starting with a discussion that deconstructs the victimizing and individualizing discourses that medicalize children's problems when young people have been exposed to experiences of adversity, the module focuses on the meaning of empowerment, resilience and beneficiaries' personal and collective potential for recovery. Participants are encouraged to shift their perspective from a deficit-focus approach to care to a strengths-based reframing of what beneficiaries have already and what they still need.
- *Module 2: Psychological trauma in children and its interaction with multilevel developmental processes*. This module provides a comprehensive summary of psychological trauma and the underlying mechanisms through which trauma affects the identity formation of beneficiaries and their functioning. Topics covered include past memory, present cognitions, emotional and social well-being and future self-projection (48, 67). While the science is presented as objective, its application to each specific context is the focus of discussions, with a heavy emphasis on local reactions to adversity and pathways to healing.
- *Module 3: Identification, prevention and appropriate responses to beneficiaries who have experienced adversity*. This module shares with trainees new tools that they may want to adapt for their work with children and families at risk. For the sake of organization, they are gathered under Grotberg's (78) three principles of resilience-building: I have, I can, I am. These activities are not intended to be used as a pre-packaged curriculum, but are more like ingredients in a grocery store which can be assembled in any number of different combinations to produce a meal (in this case, a localized curriculum) [see (79)].
- *Module 4: Self-confrontation and critical reflection on relationships that support resilience*. Trainees are asked to create meaning out of their past experience as a service provider and what it will mean to be a Tutor of Resilience with their target beneficiaries. Issues of power related to who decides which interventions are best, and the bias that trainees bring to their work because of their social and economic backgrounds are also discussed in order to prepare workers for integration with the communities where beneficiaries reside. Finally, the ToR resilience-enabling principles are shared with trainees.
- *Module 5: Monitoring the implementation of the ToR program*. A monitoring plan is developed and refined with participants. Assessment tools are trialed to identify the most appropriate ways to monitor progress by community participants. Periodic assessments throughout the ToR implementation process ensure that trainees are becoming comfortable with the model and the transformation in perspective it promotes.

A combination of didactic presentations, hands-on interactive exercises, and case studies are employed during the capacity building stage of program implementation in order to model for trainees different ways they can develop interventions for children and their families. Each module shares some of the relevant science on related topics but no specific activities are suggested as interventions. Participants are encouraged to reflect on the principles of effective practice and how these can be adapted to the needs detected in the first phase of the work with the goal of designing activities that reflect the principles trainees are learning.

### Phase 3: The Action Plan Design

At the end of the initial training, participants are required to design a Tutor of Resilience Action Plan to be implemented with their target beneficiaries and explain how their planned intervention will reflect resilience-enabling principles discussed during the workshop. The Action Plan is then submitted for review by the workshop facilitators who work individually with each trainee to refine the intervention if necessary.

### Phase 4: Follow-Up

With the assumption that programs need constant refinement and trainees need support with implementation as new challenges arise, a 2-day follow-up workshop is held within 6 months of the first workshop. Based on Kirkpatrick's model of training evaluation (80), which focus on four levels of training evaluation criteria–reactions, learning, behavior, and results–the second workshop focuses on the following themes:

1. *Program refinement*. Trainees reflect on the strengths, weaknesses, advantages and disadvantages of the ToR model and the interventions which they developed and lessons learned during the first period of program implementation.
2. *On-going evaluation and scaling up*. Trainees design a new Action Plan for their ToR program that will be executed with another group of community beneficiaries, incorporating what they learned from the first iteration of their model intervention. This second Action Plan is then scaled up where possible for broader implementation across the target community. This stage of the process also includes ongoing evaluation to assess fidelity to the principles of the ToR model.

### Phase 5: Closure

At the end of the project members of the ToR facilitation team conduct a two-day meeting with project staff (local co-facilitators who will be responsible for continuing to support the trainees in the field as they deliver services to community beneficiaries). This meeting is intended to identify lessons learned from the project as a whole and to review the ToR principles and approach in order to refine its use in other settings where MHPSS programming is required. In particular, those attending the meeting are invited to reflect on their previous experiences as protection actors and what they consider fundamental to providing an effective resilience-focused intervention in settings like theirs. As part of a knowledge mobilization strategy, these reflections from the field are later shared with external stakeholders working in fields like child protection and gender-based violence in humanitarian settings. Recommendations for refinement of the model and how best to assess outcomes are also discussed during this final meeting, with input drawn from the periodic assessments carried out with trainees. Assessment tools are further refined to make them easier to employ in poorly resourced settings like those that occur during humanitarian crises (66).

## Case Study: The Tutor of Resilience Model With Syrian and Lebanese Children in the QUDRA PROGRAM

In 2018, the ToR Model was implemented in Lebanon under the project title Qudra with funding from the European Union Regional Trust Fund in Response to the Syrian Crisis and the German Federal Ministry for Economic Cooperation and Development (BMZ). Their goal was to implement innovative approaches to strengthen the resilience of Syrian refugees, internally displaced persons (IDPs) and host communities in Jordan, Lebanon, Turkey and in the Kurdistan Region of Iraq. In Lebanon, coordination for the program was provided by Expertise France (EF), an international non-governmental organization (NGO) funded by France.

Expertise France commissioned the Tutor of Resilience model to be provided to the Lebanese Ministry of Social Affairs (MOSA) and its educators, social workers and NGO staff in charge of psycho-educational care for Syrian migrant children and Lebanese children living in situations of hardship in eight social development centers (SDC) throughout Lebanon.

Indeed, Syrian children are at heightened risk for psychiatric distress (66, 81) and developmental problems (82) due to their histories of exposure to potentially traumatizing events (83, 84) and the daily hassles and prejudice they experience in host countries (85, 86) which hinder their efforts for integration. Lebanese children taking part to the program belong to host communities that were also experiencing difficulty accessing services, in particular public sector healthcare (87) which has become strained as it responds to the needs of a growing number of refugees.

*Five Phases of Implementation:* Initial review of current practices by the local service coordinators was done and a need analysis carried out to identify the psychosocial needs and challenges experienced by service providers when dealing with the Lebanese and Syrian target communities. This assessment included the lack of shared framework and standards for how to build a helping relationship with vulnerable beneficiaries and the need for tools and interventions tailored to Syrian and Lebanese children exposed to adversity. A context-specific ToR capacity building workshop was then developed and delivered to 75 practitioners nominated for the training from their SDC. After this, the program was implemented with 641 Syrian and Lebanese children aged 7–17 in the eight SDC. Beneficiaries received either a 2-month ToR program or a 5-month ToR program, both designed and delivered by local trainees. By offering two different lengths of ToR, it was possible to evaluate the benefits of different program lengths for this population. Children were assessed before each program began (T1) and at the end (T2) to assess change in resilience and mental health. A 2-day follow up meeting was held with ToR trainees 5 months later in early 2019.

**Figure 2:**
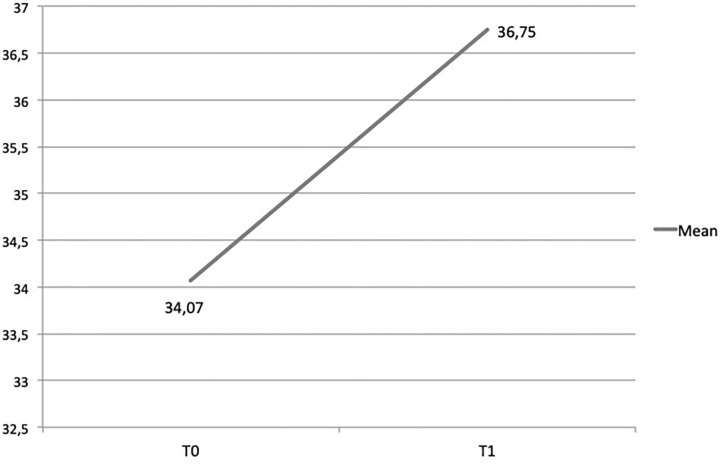
Trainees' knowledge of resilience and its application to psychosocial interventions.

**Figure 3:**
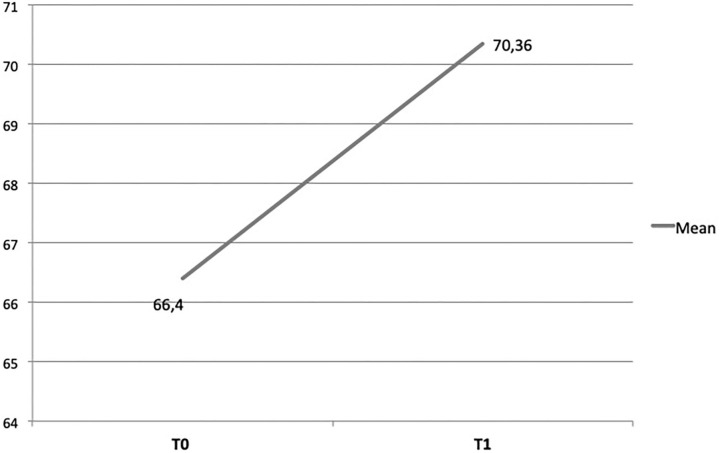
Change of trainees' self-efficacy paralleled with the ToR.

A program evaluation conducted with the QUDRA trainees showed:

1. *High levels of satisfaction with the capacity building workshop*. A Delphi exercise with a three-point Likert scale was used with practitioners at the end of the first capacity building workshop to rate the core components of the training, as well as the perceived usefulness of the ToR model (from 1 = a weak aspect of the program to 3 = a highly salient aspect of the program) to their interactions with beneficiaries. Results showed high rates of perceived usefulness: interaction and dialogue (M = 2,63; SD = 0.49); understanding of beneficiaries' problems (M = 2,60; SD = 0.55); strengthening beneficiaries' resources (M = 2,61; SD = 0.52). A final open question asked practitioners which part of the training they considered more relevant. Participants emphasized the core principles transmitted during the training, as these could guide them in tailoring activities for their target beneficiaries.
2. *Better understanding of resilience paradigm and its application to PSS*. Using a pre- and post-training questionnaire that included questions about trainees' knowledge of resilience and its application to psychosocial interventions, a Wilcoxon Signed Rank test showed that the resilience knowledge scores significantly improved for participants [*z*_(72)_ = −5.01, *p* < 0.001] (see Figure 2).
3. *Increase in practitioners' self-efficacy*. Trainees were assessed for their capacity to master the specific techniques that are unique to the program (e.g., “It's difficult for me to focus on children's resources rather than on their problems;” “I can apply my knowledge and skills when developing and implementing a resilience-focused intervention”) as well as more general aspects of program delivery (e.g., “I make children feel safe;” “I can still cope even when I feel helpless in difficult situations”). A Wilcoxon Signed Rank test showed that the practitioners' self-efficacy scores significantly improved [*z*_(36)_ = −4.78, *p* < 0.001) (see Figure 3).
4. *Increased awareness of ways to build program beneficiaries' resilience*. During the follow-up workshop, trainees reflected on their successful program designs that enhance children's resilience. These included:
  - Widening the human service workers' point of view to consider both children's impairments and their capacity to heal.
  - Challenging people's self-constructions as victims by working with their peers, caregivers and communities to better understand the impact of adversity on behavior and the resources communities have to help beneficiaries enhance their resilience while encouraging a future orientation.
  - Tailoring activities to local community settings to enhance social and emotional competencies in contextually relevant ways by including songs, stories and dances that belong to the beneficiaries' culture and by exploring the language they use to name emotions.
  - Improving relationships with both peers and family members by valorising cultural differences, creating the social cohesion necessary for sustaining mental health and social functioning, and facilitating communication between children and their families.
5. *Changes to how they conduct their practice*. A qualitative study was used to explore the changes perceived by trainees in their way of working with their target beneficiaries since implementing the ToR model. A body map research method was employed during the follow-up workshop with 36 trainees. Participants were asked to reflect on changes in their knowledge, behavior and attitudes before and after the implementation of the ToR model.
  - Body mapping is a qualitative, participatory research method to produce and disseminate knowledge about personal experiences (88), helping participants interpret, give meaning to, and make sense of their experiences (89–91). The technique has been shown to mitigate the influence of researcher bias on trainees' experiences and create a context for participants, who may feel disempowered or experience language barriers, to communicate their experiences (92).
  - Thirty-six trainees participated. All were involved in the capacity building, action plan design and the direct implementation of the ToR model with target beneficiaries. The data collection was conducted during the follow-up meeting by two ToR facilitators. Participants were asked to trace a life-sized image of their body onto a piece of paper and to reflect on the changes they detected in their knowledge, behavior and attitudes, arising from the implementation of the ToR model and draw or record these changes through words and images. Both positive or negative changes could be recorded. In particular, participants were asked how their training had changed them personally:
    ° How they think about beneficiaries (changes to their body map's head indicative of a change in cognitions).
    ° How they see beneficiaries (changes to their body map's eyes indicative of a change in perception).
    ° How they listen to beneficiaries (changes to their body map's ears indicative of a change in their attention).
    ° How they communicate with beneficiaries (changes to their body map's mouths indicative of a change in self-expression).
    ° The activities they do with beneficiaries (changes to their body map's hands indicative of a change in how they enact their relationships).
    ° Their sense of the future for beneficiaries and where beneficiaries will end up (changes to their body map's legs indicative of a change to future orientation).
  - Participants reported that ToR training and implementation led them to consider the beneficiary not only as an aid recipient, but also as an actor in the process of finding their own supports, and whose perspective needs to be taken in consideration. Furthermore, greater consideration was noted for the significant relationships in a child's life (i.e., friends, caregivers, educators) which may contribute to the child's resilience. Specifically, comments by trainees emphasized the core themes of the ToR model. For example:
    ° “I always think about my beneficiaries a lot and I remember I used to think about what would have been best for them. The Tutor of Resilience model has completely changed my perspective by changing my way of thinking about beneficiaries, so I now view things from their perspective and no longer impose my own”.
    ° “I have learnt to look at the whole picture. Sometimes children's dark side, the one made up of sufferance and difficulties, easily emerged but now I know that I must not stop there, I have to keep on searching for children's bright side, the one made up of resources and opportunities”.
    ° “The Tutor of Resilience model has changed my way of working with children. This transformation was not simple because it required changing my consolidated PSS model and adjusting to it. In fact, Tutor of Resilience training does not impose rules or protocols; rather, it frees the social worker to implement the model with creativity and innovation. While this freedom scared me in the beginning, it also gave me the opportunity to make use of my inventiveness and uniqueness while working with the children, his/her caregiver and and no longer feel like a mere executor. I don't just act as, but truly am, a tutor of resilience for children.”
  - Participants also described challenges implementing ToR. These included: insufficient time to implement the program, particularly for the trainees conducting the two-month trial; the initial workload in designing the action plan and in tailoring the activities related to the ToR principles; difficulties for trainees who have other jobs to take 4 days to attend the capacity building; difficulties for some trainees who have long experience in child protection to acquire the new perspective proposed by ToR; too little involvement of children's caregivers who, when invited, appeared reluctant to participate in the ToR sessions; the lack of appropriate spaces for the activities, where children could feel safe and comfortable to share their thoughts/emotions; and difficulty for some children to understand the items which were part of the assessment protocol. These reflections from the field have contributed to the refinement of the ToR program, which has then been shared with Lebanese and international stakeholders working in other settings where MHPSS programming is required.

## Implications

The ToR model responds to the need for multiple contextually and culturally tailored approaches to enhance the resilience of populations exposed to adversity. Populations distinguished by cultural, economic, religious and political differences require diverse interventions to promote well-being in stressed environments. This process can be facilitated by formal service providers who can enhance individual's resilience over time by offering a safe, stable, and encouraging professional relationships. There are several implications of this approach for program design and delivery that rely on the key principles of MHPSS work in humanitarian settings (1):

1. *Using a resilience paradigm as the frame of reference in MHPSS interventions is highly recommended*. Resilience is not a feature of the individual alone, but of the individual in multiple social and physical ecologies (7, 23). Within this framework, individual qualities associated with coping under adversity are activated to the extent there is capacity in the child's environment to facilitate processes that protect against risk and promote positive development. When a resilience paradigm informs interventions the focus of programming shifts from changing individuals to making social and physical ecologies more facilitative of positive growth and psychological development.
2. *Resilience approaches encourage practitioners to work simultaneously to reduce risks and strengthen protective factors*. Programs leaders are encouraged to take more active responsibility for self-improvement (72) by increasing their essential skills as resilience-enablers while engaging program participants in a similarly transformative process. This transformation heightens trainees' attention to beneficiaries' agency, considered a fundamental protective factor for the healing process.
3. *MHPSS interventions should aim at building on existing supports, strengthening longer term capacities for ongoing support and promoting local ownership of interventions*. Interventions that employ universal approaches with little contextual sensitivity and do not build on the expertise of local professionals should be avoided.
4. *Participatory training is a non-formal, ongoing education process which activates both trainers and learners with a shared set of goals*. The sharing of experiences by learners and the trainers in relation to the core principles transmitted during the training and the co-design of a shared action plan leads to a clearer understanding on culturally and contextually specific ways to mitigate risks and enable access to resilience-promoting resources.

The twin track approach proposed in the ToR model for psychosocial care highlights the importance of offering training for local mental health care providers that gives them both opportunities to reflect on their attitudes toward children and families in humanitarian crises and the tools necessary to create evidence-informed resilience-enabling practices tailored to the varied contexts where they work.

## Data Availability Statement

The raw data supporting the conclusions of this article will be made available by the authors, without undue reservation.

## Ethics Statement

The studies involving human participants were reviewed and approved by Ethics Committee of the Department of Psychology of the Catholic University of the Sacred Heart. The patients/participants provided their written informed consent to participate in this study.

## Author Contributions

FG and AC designed the model and wrote the manuscript. MU supervised it and make substantial contributions. All authors contributed to the final manuscript.

## Conflict of Interest

The authors declare that the research was conducted in the absence of any commercial or financial relationships that could be construed as a potential conflict of interest.

## Footnotes

^1^For this discussion of the model we have focused on its application to programming for children, youth and families in humanitarian settings.
